# The Pathological Anatomy of Laryngo-tracheal Phthisis

**Published:** 1882-07

**Authors:** 


### THE PATHOLOGICAL ANATOMY OF LARYNGO-TRACHEAL PHTHISIS.

Dr. John N. Mackenzie read a paper before the Medical and Chirurgical Faculty of Maryland, of which the following abstract is published in the *Maryland Medical Journal* :

He considered first *tumors of the larynx and trachea.* The author began by stating that papillary growths are common in laryngo-tracheal phthisis, being probably due to chronic hyperplastic inflammation of the connective tissue in the immediate neighborhood of tubercular ulceration. But upon diligent search he had been unable to find any record of well-defined, isolated tubercular tumors of the larynx and trachea. The two following cases of the latter, however, had come under the author's observation in the Rudolph Hospital in Vienna. The post-mortems were made by Dr. Chiari, and the specimens referred to the author for microscopic examination. The first, a *tubercular tumor of the trachea,* was taken from the body of a man who died of carcinoma of the stomach. Secondary cancerous deposits were found in the liver, kidneys, spleen, and other organs. The lungs, however, were tubercular. The pharynx, larynx and trachea were free from inflammation and ulceration. The bronchial and retro-tracheal glands were enlarged and caseous. On the posterior wall of the trachea, three-fifths of an inch above the bifurcation, was a circumscribed tumor the size of a bean. A similar growth was found in the pericardium. The former, which seemed to arise in the submucous connective tissue, was found under the microscope to consist of distinct miliary tubercular nodules implanted in hypertrophied connective tissue, the majority occupying the deeper layers of the mucous and submucous tissues. They exhibited all grades of degenerative change, some being so far advanced in caseation that nothing remained except the cellular wall. The tissues of the trachea in the neighborhood presented no remarkable change. The nodule in the pericardium showed a similar histological structure. All the other diseased organs were shown by the microscope to be cancerous.

Second, *tubercular growth of the vestibulum laryngis.* The patient died of pulmonary phthisis. The whole of the upper compartment of the larynx was covered with small nodular growths lying beneath the mucous membrane and about the size of a split pea ; each merged into its neighbor so as to form a continuous growth. The granular condition ceased abruptly at the free borders of the ventricular bands. There was no ulceration visible. Microscopic examination showed a similarity in situation and appearances to the growth above described. The deepest seated nodules were farthest advanced in caseation. The tubercles lay in close proximity to the blood-vessels, compressing them in one or two places. The larynx was, with this exception, healthy. The lungs contained tubercular cavities.

The author next considered *tuberculosis of the laryngeal muscles.* In 1877, Fraenkel examined twelve larynges, some apparently healthy, others presenting ulceration. He found constant anatomical changes, i.e. atrophy of the striated muscles. This was due in some cases to the pressure of new-formed cells in the connective tissue between the primitive bundles.

Posadsky confirmed these observations except the interstitial connective tissue change, and showed similar changes in the muscles of the extremities, intercostals and diaphragm. Heintze is unable to confirm these statements, but thinks that tubercular infiltration of the tissues is sufficient to explain the anomaly in the voice. This observer discovered tubercle imbedded in the muscular tissue of the deeper laryngeal structures, an extreme rarity according to him. Dr. Mackenzie then related a case in which he had made similar observations to those last recorded. In a patient dead of pulmonary phthisis, with cavities in both lungs, several ulcers on epiglottis and adjacent parts presented microscopically the usual appearances of tubercular infiltration and ulceration. To determine the condition of the muscular tissues, sections were made through the arytenoid cartilages and the muscles attached to their bases. Distinct miliary tubercles were found deposited in the substance and between the muscular fibres, and the fibres themselves were altered. The tubercles were imbedded deeply in the muscles near their insertion into the arytenoid cartilages, and wholly unconnected with the tubercular infiltration of the mucous membrane covering the larynx in that situation. They produced ampulliform dilatations of the fasciculi, in the midst of which they lay. The central fasciculi apparently passed through their substance or ended abruptly at it in a mass of small round cells. The tubercles were typical. Sfurounding the fasciculi was found an abundant round-cell infiltration. The most noticeable changes in the primitive bundles themselves was increase in the number and size of the muscle cells. In some places the striation was indistinct or wanting, or the muscular tissue converted into a granular detritus.

The author was inclined to regard the granulo-fatty changes in the muscular fibres as the result of disturbances of nutrition induced by chronic inflammation rather than as an integral part of the tubercular process.

